# Bilayer Dense‐Porous Li_7_La_3_Zr_2_O_12_ Membranes for High‐Performance Li‐Garnet Solid‐State Batteries

**DOI:** 10.1002/advs.202205821

**Published:** 2023-01-20

**Authors:** Huanyu Zhang, Faruk Okur, Claudia Cancellieri, Lars P. H. Jeurgens, Annapaola Parrilli, Dogan Tarik Karabay, Martin Nesvadba, Sunhyun Hwang, Antonia Neels, Maksym V. Kovalenko, Kostiantyn V. Kravchyk

**Affiliations:** ^1^ Laboratory of Inorganic Chemistry Department of Chemistry and Applied Biosciences ETH Zürich Zürich CH‐8093 Switzerland; ^2^ Laboratory for Thin Films and Photovoltaics Empa Swiss Federal Laboratories for Materials Science & Technology Dübendorf CH‐8600 Switzerland; ^3^ Laboratory for Joining Technologies & Corrosion Empa ‐ Swiss Federal Laboratories for Materials Science & Technology Dübendorf CH‐8600 Switzerland; ^4^ Center for X‐Ray Analytics Empa ‐ Swiss Federal Laboratories for Materials Science & Technology Dübendorf CH‐8600 Switzerland

**Keywords:** Li_7_La_3_Zr_2_O_12_, Li‐garnet solid‐state batteries, lithium, membranes, solid‐state electrolyte

## Abstract

Li dendrites form in Li_7_La_3_Zr_2_O_12_ (LLZO) solid electrolytes due to intrinsic volume changes of Li and the appearance of voids at the Li metal/LLZO interface. Bilayer dense‐porous LLZO membranes make for a compelling solution of this pertinent challenge in the field of Li‐garnet solid‐state batteries (SSB). Lithium is thus stored in the pores of the LLZO, thereby avoiding i) dynamic changes of the anode volume and ii) the formation of voids during Li stripping due to increased surface area of the Li/LLZO interface. The dense layer then additionally reduces the probability of short circuits during cell charging. In this work, a method for producing such bilayer membranes utilizing sequential tape‐casting of porous and dense layers is reported. The minimum attainable thicknesses are 8–10 µm for dense and 32–35 µm for porous layers, enabling gravimetric and volumetric energy densities of Li‐garnet SSBs of 279 Wh kg^−1^ and 1003 Wh L^−1^, respectively. Bilayer LLZO membranes in symmetrical cell configuration exhibit high critical current density up to 6 mA cm^−2^ and cycling stability of over 160 cycles at a current density of 0.5 mA cm^−2^ at an areal capacity limitation of 0.25 mAh cm^−2^.

## Graphical abstract

A tape‐casting method for fabricating bilayer dense‐porous Li_7_La_3_Zr_2_O_12_ (LLZO) membranes based on sequential tape‐casting of the porous and dense layers is presented. The approach enables i) to produce LLZO membranes with minimal thicknesses of both the dense (8 µm) and porous (32 µm) layers and ii) mitigates the issues of the delamination of porous and dense LLZO layers.

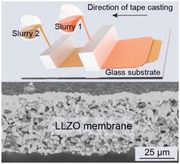

## 1 Introduction

Currently, the replacement of liquid Li‐ion electrolytes with their non‐flammable and non‐toxic solid counterparts based on Li_7_La_3_Zr_2_O_12_ (LLZO) with the garnet‐type structure is pursued as a compelling approach to improve the energy density, cycling stability, and safety of Li‐ion batteries.^[^ ^1^, ^2^, ^3^, ^4^, ^5^ ^]^ The initial studies of Werner Weppner on the exploration of garnet type LLZO solid‐state electrolytes (SSE)^[^ ^6^ ^]^ have now evolved into a broad research field,^[^ ^7^ ^]^ encompassing all aspects of underlying electrochemistry of Li‐garnet solid‐state batteries (SSB): the LLZO/Li interface,^[^ ^8^, ^9^, ^10^ ^]^ the electrochemical voltage window of LLZO,^[^ ^11^, ^12^, ^13^ ^]^ and compatibility of LLZO with current cathode chemistries.^[^ ^14^, ^15^ ^]^ Until now, however, the performance of Li‐garnet SSBs has not come close to meeting commercial requirements. Li metal anodes combined with dense LLZO ceramics are characterized by low cycling stability at current densities of >0.5 mA cm^−2^ and areal capacities of >0.5 mAh cm^−2^.^[^ ^16^, ^17^, ^18^, ^19^, ^20^, ^21^, ^22^ ^]^ Under these electrochemical conditions, the electrodeposited Li penetrates rapidly into the LLZO SSE forming dendrites that eventually short‐circuit the cell.^[^ ^23^ ^]^

**Figure 1:**
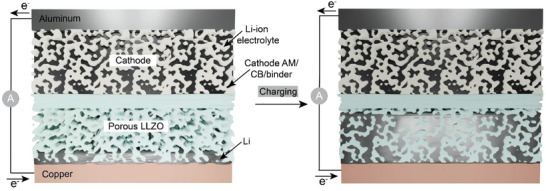
Schematics of the charging process of Li‐garnet solid‐state batteries based on dense/porous LLZO membrane.

In the context of mitigating the issues associated with Li dendrites, employment of a vastly different design of the LLZO SSE based on a dense/porous LLZO microstructure has been recently proposed (**Figure** **1**).^[^ ^24^, ^25^, ^26^, ^27^ ^]^ Such approach promises to mitigate dynamic volume changes of the Li anode and the formation of voids, both of which are assumed to be major causes of Li dendrites. On the one hand, Li can be stored in the pores of the LLZO scaffold during Li deposition, thereby avoiding dynamic changes in cell volume. On the other hand, the formation of voids during stripping can be mitigated by the larger surface area of the LLZO/Li interface in the scaffold compared to dense LLZO ceramics. The upper dense part of the membrane could act as an additional protective layer that mitigates potential short circuits during cell charging. To date, the works on porous LLZO membranes have been published mainly by the group of Wachsman.^[^ ^24^, ^28^, ^29^, ^30^, ^31^, ^32^, ^33^ ^]^ The developed approach is based on tape‐casting of porous and dense LLZO tapes followed by their compressing, drying, and sintering. Considering the necessity of the cathodic or anodic substrates when employing other methods (wet‐chemical, PVD, CVD), the tape‐casting technology that enables the fabrication of free‐standing membranes is considered one of the most prominent fabrication strategies for the industry.^[^ ^34^, ^35^ ^]^ Tape‐casting approach, nevertheless, still faces a number of obstacles before it can be practically deployed. Some of the major issues lie in maintaining the flatness of the membranes, the contact between dense and porous layers, and the proper Li stoichiometry after sintering. Moreover, with respect to the achievable energy densities of Li‐garnet SSBs, minimizing the thickness of the dense and porous layers is of paramount importance.^[^ ^36^ ^]^ The achievable energy density of Li‐garnet SSB comprising reported LLZO membranes is estimated to be 150–250 Wh kg^−1^ and 390–820 Wh L^−1^, based on the thickness of the porous and dense layers (discussed below).

In this work, we present a novel tape‐casting method for fabricating LLZO membranes based on sequential tape‐casting of the porous and dense layers. The presented approach enables i) to produce LLZO membranes with minimal thicknesses of both the dense (8–10 µm) and porous (32–35 µm) layers and ii) to mitigate the issues of the delamination of porous and dense LLZO layer after the sintering. We demonstrate that developed LLZO membranes in symmetrical cell configuration exhibit a high critical current density (CCD) of up to 6 mA cm^−2^ and low overpotentials of 14–16 mV at a current density of 1.0 mA cm^−2^. Additionally, the symmetrical cells delivered high Li plating/stripping cycling stability of over 160 cycles at a current density of 0.5 mA cm^−2^ with an areal capacity limitation of 0.25 mAh cm^−2^. The electrochemical performance of dense/porous LLZO membranes is also assessed with a LiFePO_4_ (LFP) cathode. Li/LLZO/LFP full cells deliver stable capacities of around 0.4 mAh cm^−2^ at 0.1 C rate (a current density of 0.05 mA cm^−2^) for 30 cycles.

## 2 Results and Discussion

### 2.1 The Preparation of Li_7_La_3_Zr_2_O_12_ Membranes

In short, bilayer porous‐dense LLZO membranes were prepared in a few steps that include: i) Preparation of slurries for the fabrication of porous and dense LLZO layers, ii) their sequential tape‐casting on a glass substrate, iii) drying of the deposited layers, iv) pilling off the LLZO tape from the substrate, v) followed by their de‐binding (600 °C, air), and vi) sintering (1100 °C, N_2_). Both slurries were prepared using similar procedure by ball milling of LLZO, Li_2_CO_3_, plasticizer, surfactant, binder, and solvent. In the case of preparation of the slurry for porous LLZO layers, polymethyl methacrylate (PMMA) pore‐formers were additionally used.

**Figure 2:**
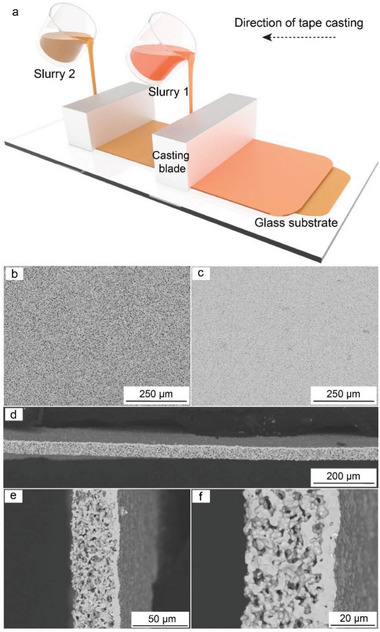
a) Schematic of sequential tape‐casting of porous and dense LLZO layers on the glass substrate. Top‐view SEM images of b) porous and c) dense layers of LLZO membranes. d–f) Cross‐section SEM images of LLZO membranes after sintering.

It was found that sequential tape‐casting of porous and dense layers (**Figure** **2**a) allows achieving high adhesion of the two layers. As shown in Figures S1, Supporting Information, and Figure 2d–f, no delamination was observed between the two layers along the entire interface of the prepared membranes after both de‐binding and sintering. Notably, we found that the previously reported methods for producing bilayer LLZO membranes, based on i) pressing pre‐dried porous and dense LLZO tapes, followed by their de‐binding and sintering, or ii) sintering of superimposed porous and dense membranes after de‐binding were far less reproducible. Specifically, cross‐section scanning electron microscopy (SEM) images of sintered membranes revealed numerous delaminated areas between both porous and dense layers (Figures S2 and S3, Supporting Information).

**Figure 3:**
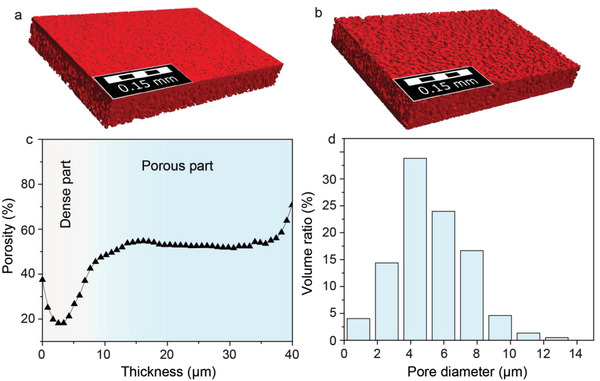
a) Top (dense) and b) bottom (porous) view of reconstructed micro‐computed tomography slices of dense/porous LLZO membranes. c) The computed porosity of the LLZO membrane along the Z‐axis (thickness). d) Size histogram of pores inside of porous LLZO layer. Detailed information on the analysis of the reconstructed 3D images of LLZO membranes can be found in Section 4.

The characterization of the produced membranes by SEM (Figure 2b–f) and X‐ray computed tomography (**Figure** **3**) confirmed the formation of porous LLZO microstructure with a pore size of 2–10 µm. The average porosity was ≈50–55 vol% within the whole thickness of the porous layer of LLZO membrane (Figure 3). Importantly, as follows from the analysis of X‐ray tomography images of LLZO membranes, the porous layer contains only open‐pore channels (Table S1, Supporting Information). As to the dense LLZO layer, top‐view SEM, and X‐ray tomography images of LLZO membranes indicate the formation of a non‐porous, pin‐hole‐free, and continuous dense LLZO layer (Figure 2c–d and Figure 3a,b).

X‐ray diffraction (XRD) analysis confirmed the formation of a phase‐pure cubic LLZO structure (Figure S4, Supporting Information, the space group, Ia$\bar{3}$d, *a* = 12.9622(2) Å, *V* = 2177.89 Å^3^, ICSD 235 896). Importantly, our preliminary experiments revealed that the addition of Li_2_CO_3_ as an additive to compensate Li‐losses during de‐binding and sintering is paramount for obtaining an impurity‐free cubic LLZO phase. Thus, it was identified that in the case of using LLZO powder without the addition of Li_2_CO_3_, the sintered LLZO membranes contained large quantities of La_2_Zr_2_O_7_ (LZO).

**Figure 4:**
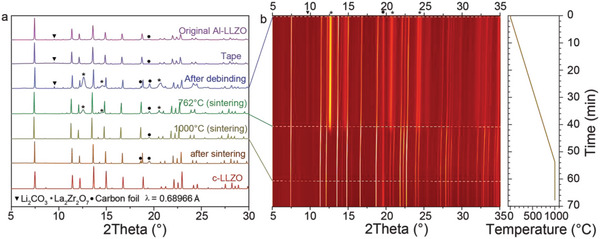
In situ SXRD measurements of LLZO membrane during heat‐treatment to 1000 °C. a) SXRD patterns of LLZO membrane at different stages of the heat‐treatment. XRD patterns of as received LLZO powder and tape‐casted LLZO tape are given for comparison. b) Measured SXRD map of LLZO membrane during heat‐treatment. Right side: Temperature profile of the heat‐treatment.

For comprehending the chemical transformations that occur during de‐binding and sintering of tape‐casted LLZO tapes, the tapes were analyzed by in situ synchrotron X‐ray diffraction (SXRD) during their heat‐treatment up to 1000 °C. It has also been revealed that multiple chemical processes occur (**Figure** **4**). First, the de‐binding causes the formation of a relatively large quantity of the LZO phase, as follows from the comparison of as‐prepared LLZO tape and de‐binded LLZO membranes. Upon further increase of temperature to 760 °C, however, the intensity of LZO reflections starts to disappear, resulting in the formation of a solely cubic LLZO structure. This process accompanies by the disappearance of the Li_2_CO_3_ peak at ≈716 °C, which is most probably associated with the melting of Li_2_CO_3_. Notably, the thermal decomposition of Li_2_CO_3_ has also been confirmed by TGA‐DSC‐MS measurements (Figure S5, Supporting Information). Specifically, it was identified significant weight losses of tape‐casted LLZO tapes at ≈600 °C, which was accompanied by the appearance of MS CO_2_ peak at m/z = 16, 28, and 44. This process intensifies at 730 °C, followed by a sharp decrease of LZO phase at 760 °C, and the total disappearance of LZO phase at 860 °C.

Importantly, we noticed that membranes with higher Li_2_CO_3_ content had higher density as compared with lower or no Li_2_CO_3_ content after sintering under the same conditions (Figure S6, Supporting Information). This observation is in line with the reported impact of Li_2_CO_3_ on the densification of LLZO pellets.^[^ ^37^, ^38^ ^]^ We assume that this effect is related to the melting of Li_2_CO_3_, which allows for initiating the sintering process at lower temperatures. The impact of Li_2_CO_3_ is similar to that observed when LiF is used as a sintering agent for sintering Al_2_O_3_‐based ceramics.^[^ ^39^, ^40^, ^41^ ^]^ LLZO ceramics without additional Li_2_CO_3_ requires a higher temperature and longer time for sintering and potentially causing huge Li‐loss.

### 2.2 Surface Characterization

**Figure 5:**
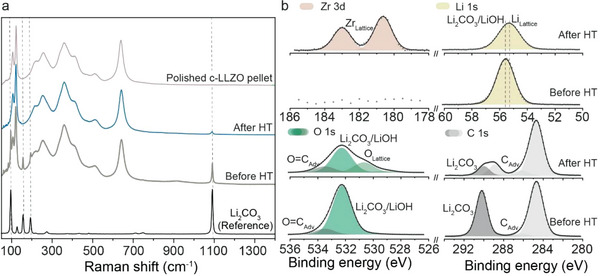
a) Raman spectra and b) charge‐corrected Li 1s, Zr 3d, C 1s, and O1s XPS spectra of LLZO membranes before and after heat‐treatment (HT) at 600 °C for 1 h. Raman spectrum of polished c‐LLZO pellet and Li_2_CO_3_ powder are given for comparison.

To solve the issue of poor wettability of the sintered LLZO scaffolds by lithium metal, caused by the presence of LiOH and Li_2_CO_3_ on the LLZO surface, the membranes were annealed at 600 °C in Ar atmosphere. The presence of LiOH and Li_2_CO_3_ has a decisive impact on the electrochemical performance of Li‐garnet SSBs.^[^ ^42^, ^43^ ^]^ First, it leads to an increase in Li/LLZO interfacial resistance and consequently to high‐voltage polarization of the cell upon Li plating/stripping. Second, this triggers the formation of Li dendrites due to the inhomogeneous distribution of the applied current density. As shown by the Raman spectroscopy measurements, heat‐treatment of the sintered LLZO membranes at 600 °C for 1 h significantly reduced the amount of Li_2_CO_3_ on the LLZO surface (**Figure** **5**a). In particular, the Raman spectroscopy data showed that the Raman peaks at 95 and 1090 cm^−1^, which are associated with the presence of Li_2_CO_3_ on the LLZO surface, had almost disappeared after the heat treatment. Other Raman peaks, such as those in the 100 to 600 cm^−1^ range, which corresponds to the cubic structure of LLZO, did not change. Importantly, the heat treatment preserved the cubic LLZO structure of the membranes, as follows from their XRD patterns before and after annealing. Of note, the heat treatment of the LLZO membranes at higher temperatures of 750 and 900 °C resulted in the formation of the LZO phase due to intensified Li losses (Figure S7, Supporting Information).

Next, the surface chemistry of the sintered LLZO membranes before and after heat‐treatment was investigated using X‐ray photoelectron spectroscopy (XPS). Notably, the characterized LLZO membranes were transferred under an Ar shielding gas atmosphere to the XPS instrument, thus hindering a reaction of the highly‐reactive LLZO surfaces with air. To correct for differential charging of the insulating LLZO surfaces during XPS analysis, the recorded photoelectron spectra were charge corrected by shifting them corresponding to the difference between the adventitious C 1s peak (at the lower binding energy side of the C 1s peak envelop in the recorded XPS survey) and the recommended reference value for adventitious carbon at 284.8 eV (Figure S8, Supporting Information). Figure 5b shows the charge‐corrected Zr 3d, O 1s, C 1s, and Li 1s spectra of the sintered LLZO membranes before and after heat treatment. As follows from the analysis of O 1s binding energies shown in Figure 5b, the probed surface of the non‐heat‐treated LLZO membranes contains two O chemical states. The main O1 s component at 532.2 eV can be assigned to Li_2_CO_3_/LiOH, whereas the minor component at 533.5 eV is attributed to C = O bonding states.^[^ ^44^ ^]^ Evaluation of the C 1s and Li 1s lines for the non‐heat‐treated LLZO membranes confirms the presence of a Li_2_CO_3_/LiOH surface layer. The C 1s peak at 290.2 eV and the O 1s peak at 532.2 eV can be attributed to Li_2_CO_3_.^[^ ^18^ ^]^ The main C 1s peak at 284.6 eV can be assigned to adventitious carbon (C—C bond), which inevitably arises from the interaction of volatile organic species in the glove box (as originating from the chemical synthesis environment) with the membrane surface. Note that no Zr signal from the LLZO lattice (in the range of 178–186 eV) could be detected before the heat treatment. The probing depths for the Zr 3d, O 1s, C 1s, and Li 1s spectra recorded from LLZO using Al‐K*α* X‐ray radiation (*hν* = 1486.7 eV) are 5.5, 4.8, 5.5, and 6.6 nm, respectively.^[^ ^8^ ^]^ Hence, the Li_2_CO_3_/LiOH surface layer for the non‐heat‐treated LLZO membranes should be significantly thicker than 6 nm. After the heat treatment, the Zr peaks from the LLZO lattice (at 183.0 and 180.6 eV) could be detected. Moreover, the C 1s and O 1s spectral contributions from Li_2_CO_3_ were significantly reduced. In addition, an additional O 1s peak arises at around 530.7 eV, as attributed to O in the LLZO lattice. As discussed in ref. [8] the chemical shifts of the Li 1s photoelectron lines between the individual chemical species are relatively small (as compared to their respective intrinsic peak widths) and can therefore not be resolved unambiguously. However, a spectral contribution from Li in the LLZO lattice should give rise to an effective shift of the Li 1s peak envelop toward lower binding energies, as indeed observed after the heat treatment (the Li 1s shifts from about 55.5 to 55.3 eV). The XPS analysis clearly indicates that the Li_2_CO_3_/LiOH surface contamination layer is largely removed by the heat treatment. The remaining O 1s and C 1s signal intensities from Li_2_CO_3_/LiOH imply an effective Li_2_CO_3_/LiOH layer thickness well below 5 nm. The XPS analysis clearly shows that the heat treatment of LLZO membranes very effectively removes the Li_2_CO_3_/LiOH contamination layer.

### 2.3 Electrochemical Characterization

Aiming to determine the Li‐ion conductivity of sintered LLZO ceramics, we fabricated 38 µm thick, highly dense (95–98% of the theoretical density of 5.1 g cm^−3^) LLZO membranes. Electrochemical impedance spectroscopy (EIS) measurements revealed that the ionic conductivity of the LLZO membranes was ≈1.9 × 10^−3^ S cm^−1^ (see Figure S9, Supporting Information), which is consistent with the values of Li‐ion conductivity of LLZO pellets reported in the literature. In addition, we performed EIS measurements on Li/LLZO/Li symmetrical cells assembled with non‐heat‐treated and heat‐treated (600 °C; 1 h; Ar atmosphere) LLZO membranes. As shown in Figure S10, Supporting Information, the heat‐treatment leads to a decrease in Li/LLZO interfacial resistance from 192 kΩ cm^2^ (RT) to 116 Ω cm^2^ (600 °C). As discussed above, such difference is associated with the decrease of Li_2_CO_3_/LiOH content on the LLZO surface.

**Figure 6:**
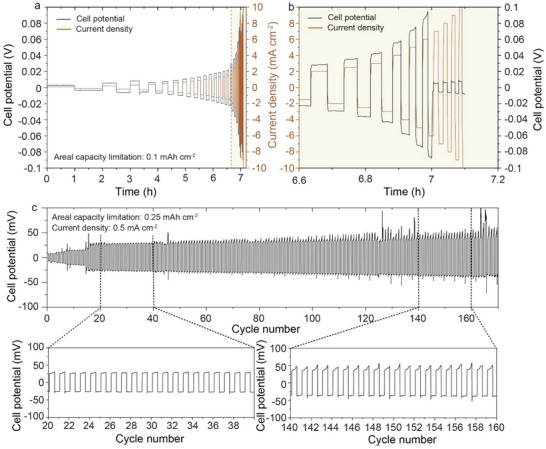
a,b) CCD voltage profiles of Li/LLZO scaffold/Li symmetrical cells measured with a capacity limitation of 0.1 mAh cm^−2^ per half‐cycle ((b) shows enraged beige area of the figure (a)). c) Voltage profiles of Li/porous LLZO membrane/Li symmetrical cell measured at a current density of 0.5 mA cm^−2^ and the areal capacity limitation of 0.25 mAh cm^−2^ per half‐cycle.

To examine the electrochemical performance of self‐standing porous LLZO membranes with respect to Li plating/stripping, symmetric Li/LLZO/Li cells were fabricated by cold isostatic pressing Li onto a heat‐treated LLZO single porous layer membrane at ≈71 MPa. This method allowed impregnation of up to ≈10 µm of Li within the 50% porous LLZO scaffold (Figure S11, Supporting Information), corresponding to an areal capacity of ≈1 mAh cm^−2^. The achievable CCD of the studied systems, that is, the current density at which the propagation of Li dendrites/filaments starts, was determined by galvanostatic cycling experiments at different current densities. Specifically, the current density was increased from 0.1 to 1.5 mA cm^−2^ with a step of 0.1 mA cm^−2^, from 1.5 to 3 mA cm^−2^ with a step of 0.5 mA cm^−2^, and from 3 to 10 mA cm^−2^ with a step of 1 mA cm^−2^, transferring the same amount of Li for each half cycle (0.1 mAh cm^−2^). The tests were performed at 60 °C without applying any stack pressure. Symmetric cells are found to have a high CCD of up to 6 mA cm^−2^ (**Figure** **6**a).

Additionally, galvanostatic cycling experiments were conducted using a capacity limitation of 0.25 mAh cm^−2^ per half‐cycle at a current density of 0.5 mA cm^−2^ without external pressure at room temperature. The typical galvanostatic voltage profiles of symmetrical cells and cross‐sectional SEM images of LLZO membrane after half‐cycle of Li plating are shown in Figure 6c and Figure S11, Supporting Information, respectively. Importantly, cells demonstrated high cycling stability over 160 cycles.

**Figure 7:**
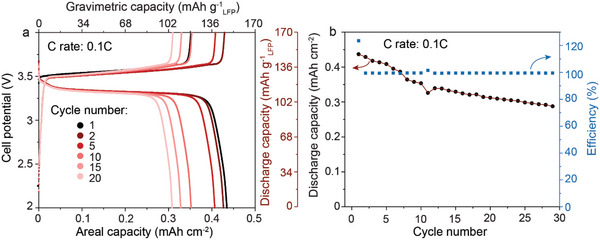
Galvanostatic charge–discharge a) voltage profiles and b) cyclic stability of LFP/LLZO membrane/Li full cells were measured at 0.1 C rate and room temperature without the employment of external pressure.

Next, we probed the electrochemical functionality of the developed bi‐layered dense/porous LLZO scaffolds in full hybrid‐type cells in combination with paste‐type LFP cathode (**Figure** **7**). In short, the cathode paste was prepared by mixing LFP (41 wt%), carbon black (13.7 wt%), and 0.3 m LiTFSI in a PY_14_TFSI ionic liquid electrolyte (45.3 wt%). The paste electrode was deposited directly on the cellulose separator (placed on the dense side of the LLZO membrane), and then covered with Al foil as a current collector. The opposite porous side of the LLZO membrane was prior infiltrated with metallic lithium by isostatic pressing at 71 MPa. The average loading of LFP active material was ≈3 mg cm^−2^. The cells were tested at room temperature and without applying external pressure. The applied currents were normalized to the surface area of the Li anode. All electrochemical measurements were performed using galvanostatic cycling within the voltage range of 2–4.2 V versus Li^+^/Li. Figure 7 shows the voltage profiles and cycling stability measurements of LFP/LLZO/Li full cell measured at 0.1 C rate. Cell delivered the capacity of LFP of ≈100–150 mAh g^−1^, which corresponds to the areal capacity of ≈0.3–0.45 mAh cm^−2^. The results demonstrate relatively low capacity retention of 65% after 30 cycles, pointing to the need for further optimization of the cathode.

Apart from the experimental results, we assessed the feasibility of employment of developed LLZO membranes in commercial Li‐garnet SSBs and compared them with previously reported LLZO membranes considering their thickness and porosity. The evaluation of the achievable gravimetric and volumetric energy densities of full cells was performed using LiNi_0.8_Mn_0.1_Co_0.1_O_2_ (NMC811) cathode due to its high discharge voltage of 3.8 V versus Li^+^/Li and gravimetric and volumetric capacities (180 mAh g^−1^ and 858 mAh cm^−3^). Briefly, the calculations were performed for a full cell consisting of a porous/dense LLZO membrane, and an NMC811 cathode with an areal capacity of 3 mAh cm^−2^, as recently reported in our work (see ref. [44, 45] for details). The anode side is designed to be anode‐free.

**Figure 8:**
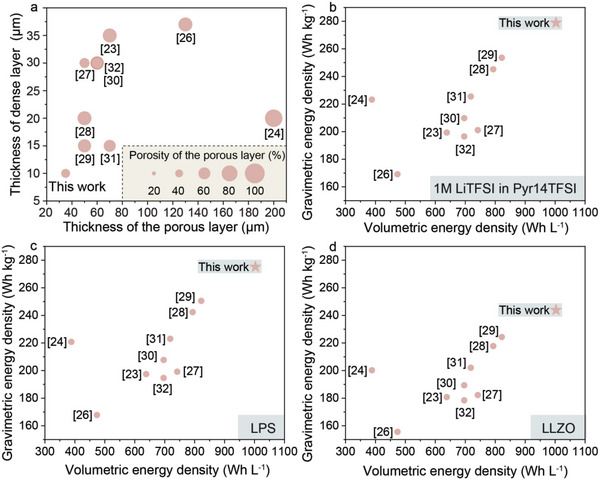
a) Summary of thicknesses of dense/porous layers and porosity of the porous layer for previously reported dense/porous LLZO membranes and the ones developed in this work. The comparison of calculated gravimetric and volumetric densities of Li‐garnet SSBs based on previously reported and developed in this work dense/porous LLZO membranes combined with b) LiTFSI‐Pyr14TFSI/NMC811, c) LPS/NMC811, or d) LLZO/NMC811 cathodes.

The cathode consisted of NMC811 active material, carbon black, pVdF binder, and Li‐ion electrolyte of different densities (1 m LiTFSI in PY14TFSI (1.7 g cm^−3^), Li_7_P_3_S_11_ (LPS) (2.0 g cm^−3^), or LLZO (5.1 g cm^−3^)) (see Figure S12, Supporting Information, for details). All other parameters used for the calculations are given in Table S2, Supporting Information. Below, we summarize the key parameters of the reported dense/porous LLZO membranes, such as the thicknesses of the dense/porous layers and the porosity of the porous layers (**Figure** **8**a), and the corresponding achievable energy densities of hypothetical full cells based on these LLZO membranes (Figure 8b). Our analysis shows that the developed LLZO membranes enable to achieve gravimetric and volumetric energy densities of Li‐garnet batteries that are superior to those based on the previously reported dense/porous LLZO structures. Specifically, the gravimetric energy densities of Li‐garnet batteries with NMC811/ionic liquid, NMC811/LPS, or NMC811/LLZO cathodes were 279, 275, and 244 Wh kg^−1^, respectively. The volumetric energy density of the hypothetical Li‐garnet batteries was determined to be 1003 Wh L^−1^. On the one hand, the calculated results clearly show the importance of minimizing the thickness of the dense layer. On the other hand, the obtained data indicate that the thickness of the porous layer should only slightly be higher than the thickness required for complete deposition of Li in the pores of the LLZO scaffold. It should be noted, however, that both the gravimetric and volumetric energy densities of non‐optimized full cells based on LFP cathode, whose electrochemical performance demonstrated in Figure 7 were 58 Wh kg^−1^ and 111 Wh L^−1^, accordingly. Such low values are primarily associated with low areal capacity of used LFP cathodes.

## 3 Conclusions

In summary, we report a facile methodology for fabricating 10‐µm‐dense/35‐µm‐porous LLZO membranes with thicknesses maximally close to those required for achieving high gravimetric and volumetric energy densities of Li‐garnet SSBs. The assessed values of the energy densities of Li‐garnet batteries composed of the developed dense/porous LLZO membranes are 279 Wh kg^−1^ and 1003 Wh L^−1^, accordingly (in combination with NMC811 cathode that impregnated with 1 m LiTFSI in PY14TFSI ionic liquid electrolyte). It has been found that for obtaining proper contact between the dense and porous layers after sintering, the use of sequential tape‐casing of the two layers on one substrate is of utmost importance. Other methods in which the dense and porous layers are tape‐casted separately are far less reproducible.

With a suite of diverse characterization methods—SXRD, Raman, and TGA‐MS—chemical and structural transformations occurring during de‐binding and sintering of LLZO membranes were unveiled. It was also found that the addition of Li_2_CO_3_ as an additive, which allows to compensate Li losses at high temperatures, is a major factor in obtaining phase‐pure cubic LLZO membranes after sintering. After removing impurities from the surface of LLZO membranes by Ar heat‐treatment at 600 °C for 1 h, their electrochemical performance was evaluated in symmetrical and full cell configurations. We determined that developed LLZO membranes exhibit a high CCD of up to 6 mA cm^−2^ and long cycling stability for 160 cycles at a current density of 0.5 mA cm^−2^ and the areal capacity limitation of 0.25 mAh cm^−2^ without the employment of external pressure. The electrochemical performance of dense/porous LLZO membranes has also been assessed with an LFP cathode. Li/LLZO/LFP full cells delivered initial areal capacities of around 0.45 mAh cm^−2^ and 65% of this capacity was retained after 30 cycles at a current density of 0.05 mA cm^−2^ (≈0.1 C). In the context of future work, we propose that subsequent research on LLZO membranes should focus on implementing scalable roll‐to‐roll sintering processes to enable eventual commercialization of Li‐garnet SSB.

## 4 Experimental Section

### Chemicals

Al‐LLZO (Ampcera, Al‐doped LLZO, 500 nm nano‐powder), PMMA spheres (2–10 µm, SUMPMMA‐S, Sunjinbs), Li_2_CO_3_ (Sigma‐Aldrich), isopropanol (Emsure), ethanol (Sigma‐Aldrich), 1‐propanol (99.5%, AcroSeal), LiTFSI (Solvionic), PY14TFSI (Iolitec).

### Preparation of Li_7_La_3_Zr_2_O_12_ Slurry and Tape‐Casted Single‐Layer Li_7_La_3_Zr_2_O_12_ Tapes

Al‐LLZO, PMMA (pore former), Li_2_CO_3_, surfactant solution, plasticizer, and solvent (5 vol% of isopropanol, 87 vol% ethanol, and 8 vol% 1‐propanol) were premixed with a spatula in a ball milling jar followed by their ball‐milling at 165 rpm for 18 h. Then, a binder solution (Polyvinyl butyral in isopropanol) was added to the suspension, which was further ball‐milled at 200 rpm for 2 h. Prepared LLZO slurry was tape‐casted onto glass substrate. After keeping prepared LLZO tapes for 1 h under ambient conditions for solvent evaporation, LLZO tape was peeled off from the glass substrate for further de‐binding and sintering. For the preparation of dense single‐layer LLZO membranes or dense LLZO layers in bilayer dense/porous LLZO membranes, the slurry without PMMA pore formers was used.

### Preparation of Tape‐Casted Double‐Layer (Porous/Dense) Li_7_La_3_Zr_2_O_12_ Tapes

Tape‐casted double‐layer (porous/dense) LLZO tapes were prepared by sequential tape‐casting of LLZO slurries with and without PMMA pore formers with the time delay of ≈60 s. Prepared LLZO tape was kept for ≈1 h under ambient conditions followed by their removal from the glass substrate for further de‐binding and sintering.

### De‐Binding and Sintering of Li_7_La_3_Zr_2_O_12_ Membranes

LLZO tapes were cut into ≈1 × 1 cm^2^ squares, which were placed between two alumina plates. Afterward, LLZO squares were treated in air to 600 °C, resulting in complete removal of used solvent (up to 150 °C), decomposition of PMMA pore formers (≈350 °C) and removal of residual organics such as binder and plasticizer (at ≈600 °C). The de‐binded membranes were placed between two graphite foils, sandwiched by two additional carbon plates and sintered at 1100 °C for 15 min under an N_2_ atmosphere. Afterward, sintered LLZO membranes were heat‐treated at 600 °C in air to remove carbon residue from the LLZO surface. This heat‐treatment procedure was followed by the additional heat‐treatment in the air‐filled glovebox at 600 °C for 1 h to remove any contamination originating from the presence of Li_2_CO_3_ or LiOH on the LLZO surface.

### Materials Characterization

SEM images were recorded using a Hitachi TM3030Plus Tabletop microscope with an acceleration voltage of 10 kV.

X‐ray computed tomography measurements were conducted on EasyTom XL Ultra 230‐160 micro/nano‐CT scanner (RX Solutions, Chavanod France). The scanner operated at 90 kV and a current of 160 µA. The samples were scanned full 360° with a rotation step of 0.2° and frame average of 10. The nominal resolution was set at 850 nm voxel size. Image reconstruction was performed using the X‐Act computed tomography software (RX Solutions, Chavanod, France). Quantitative 3D and 2D analyses of the 16‐bit TIFF format reconstructed images were performed using GeoDict software.

In situ SXRD measurements were performed at the Swiss Norwegian Beamline (SNBL), BM01, at the European Synchrotron Radiation Facility (ESRF, Grenoble, France) using the PILATUS@SNBL diffractometer (*λ*  =  0.68966 Å) in a high‐intensity beam mode (≈200 mA). The experiments were conducted under N_2_ atmosphere using custom‐made furnace,^[^ ^46^ ^]^ which was gradually heated at the ramp rate of 20 °C per min up to 1000 °C. The data acquisition time was 10 s per pattern. The 2D diffraction data from a Pilatus 2 m detector were processed using the SNBL Toolbox and BUBBLE software. The visualization of SXRD peaks during sintering was done by Modulation‐Enhanced Diffraction Viewer and Editor (Medved) software, developed by SNBL.^[^ ^47^ ^]^ TGA and mass spectrometry (MS) were performed using a Netzsch simultaneous thermal analyzer (STA 449 F5 Jupiter) coupled with a quadrupole mass spectrometer (QMS 403 D Aëolos). The samples (10 mg) were heated to 1150 °C at 450 °C h^−1^ under Ar gas flow (20 mL min^−1^) in alumina crucibles.

Raman spectroscopy measurements were conducted on a confocal Raman microscope (Horiba, LabRAM HR Evolution) equipped with Nd:Yag 532 nm laser (Cobolt SambaTM). The measured LLZO samples were sandwiched in between two thin glass slides, which were sealed inside of the Ar‐filled glovebox by epoxy glue in order to prevent their exposure to air.

XPS analysis was performed using a PHI Quantes spectrometer (ULVAC‐PHI), using the monochromated Al‐K*α* radiation (1486.6 eV; power 100 W; beam diameter ≈100 µm). The XPS spectrometer was directly connected to an Ar glovebox, enabling to transfer LLZO under Ar shielding gas. As such, air exposure of the as‐prepared and heat‐treated membranes could be prevented. XPS survey spectra were recorded with a pass energy of 280 eV and a step size of 0.5 eV. Detailed Li 1s, Zr 3d, C 1s, and O 1s regions were recorded with step size of 0.10 eV and a pass energy of 69 eV. The energy scale of the hemispherical analyzer was calibrated according to ISO 15 472 by referencing the Au 4f_7/2_ and Cu 2p_3/2_ main peaks (as measured in situ for corresponding sputter‐cleaned, high‐purity metal references) to the recommended BE positions of 83.96 and 932.62 eV, respectively. Charge neutralization during each measurement cycle was accomplished by a dual beam charge neutralization system, employing low energy electron and argon ion beams (1 V Bias, 20 µA current).

Powder XRD was measured on a STOE STADIP Dual Setup (Cu‐ and Ag‐​radiation) with Mythen Detectors in transmission mode (Cu‐K*α* irradiation, *λ* = 1.5406 Å).

EIS measurements were conducted using a frequency range of 1 MHz to 0.1 Hz with a sinus amplitude of 10 mV.

### Preparation of Symmetrical and Full Cells

Symmetric Li/LLZO/Li cells were fabricated by thermal evaporation of 200 nm metallic Li followed by cold isostatic pressing of Li foil (at ≈71 MPa for 5 min) on both sides of the LLZO scaffold. Thermal evaporation and cold isostatic pressing of Li were performed using Covap thermal evaporator (Angstrom) and PW 100 EH cold isostatic press (P/P/Weber). Isostatic pressing allowed to impregnate of up to ≈10 µm of Li at each side of LLZO scaffold, corresponding to the areal capacity of ≈1 mAh cm^−2^. It should be noted that prepared LLZO membranes are fragile and require careful handling during cell assembly.

Full cells were prepared employing bilayer porous/dense LLZO membranes. First, 200 nm metallic Li was evaporated on the porous side of the LLZO membrane, followed by the cold isostatic pressing of Li foil at ≈71 Mpa for 5 min. Afterward, a thin 20 µm cellulose separator (Mitsubishi) was placed on the dense side of LLZO membrane, and then covered by paste‐type LFP cathode and Al foil.

### Fabrication of LiFePO_4_ Cathodes

LFP (41 wt%), carbon black (13.7 wt%), and 0.3 m LiTFSI in PY_14_TFSI ionic liquid electrolyte (45.3 wt%) were mixed in a mortar to form a paste‐type LFP electrode. The loading of active material was ≈3 mg cm^−2^.

Electrochemical measurements were performed in the Ar‐filled glove box using a multichannel workstation (MPG ‐2, Bio‐Logic SAS). EIS measurements were conducted in a frequency range of 1 MHz to 0.1 Hz with a sinus amplitude of 70 mV. The sharp drop of the overpotential and resistance (Figure S13, Supporting Information) of Li/LLZO/Li symmetrical cells was used as an indication of cell short‐circuit.

### Calculations of Energy Density

The achievable gravimetric and volumetric energy densities of anode‐free Cu/LLZO/NMC811/Al full cells were calculated considering cathode with the areal capacity of 3 mAh cm^−2^, 100% of NMC811 active material utilization, average cell voltage of 3.8 V, and total weight or volume of all cell components. The cell volume was calculated in the fully discharged state, which is the state in which the battery would be assembled. The cell was assumed to consist of 2 layers of pouch‐Al foil, 20 double‐side coated cathode layers on Al foil, 20 layers of Al foil, 40 layers of LLZO membrane, and 20 layers of Cu foil. The dimensions of the battery were 5.5 cm × 8.5 cm. It was assumed that the cathode was composed of 30 vol% of electrolyte (LPS, LLZO, or LiTFSI‐PY14TFSI ionic liquid) and 70 vol% NMC811/CB/pVdF mixture, respectively. NMC811/CB/pVdF mixture based on 95 wt% NMC active cathode material, 3 wt% carbon black, and 2 wt% of the pVDF binder was considered. Additionally, it was assumed that there was no unoccupied pore volume. The thicknesses of NMC811 cathode, Al foil, Cu foils, and packaging foil were 57, 16, 12, and 50 µm, respectively. Areal weight of the packaging foil was 10.5 mg cm^−2^. The densities of all cell components can be found in Table S2, Supporting Information. In the case of calculations of energy density for experimentally demonstrated full cells based on paste‐type LFP cathode, the same battery dimensions (5.5 cm × 8.5 cm) and similar other parameters were considered. Specifically, it was assumed that the cell is composed of 2 layers of packaging foil, 20 double‐side coated cathode layers on Al foil, 20 layers of Al foil, 40 layers of 25‐µm‐thick cellulose separator impregnated with ionic liquid electrolyte, 40 layers of LLZO membrane, and 20 layers of 40‐µm‐thick Li foil. The density of the cellulose separator with ionic liquid electrolyte was 1.416 g cm^−3^.

## Conflict of Interest

The authors declare no conflict of interest.

## Supporting information

Supporting Information

## Data Availability

The data that support the findings of this study are available from the corresponding author upon reasonable request.
