# Supplementary material for: Bilayer Dense‐Porous Li7La3Zr2O12 Membranes for High‐Performance Li‐Garnet Solid‐State Batteries
Source: Adv Sci (Weinh). 2023 Jan 20;10(8):2205821. doi: 10.1002/advs.202205821 (PMC10015908; doi:10.1002/advs.202205821)
Supplement: Supplementary file 1 — Supporting Information [file ADVS-10-2205821-s001.pdf]

ADVANCED SCIENCE Open Access

Supporting Information

for *Adv. Sci.*, DOI 10.1002/advs.202205821

Bilayer Dense-Porous $Li_7La_3Zr_2O_{12}$ Membranes for High-Performance Li-Garnet Solid-State Batteries

Huanyu Zhang, Faruk Okur, Claudia Cancellieri, Lars P. H. Jeurgens, Annapaola Parrilli, Dogan Tarik Karabay, Martin Nesvadba, Sunhyun Hwang, Antonia Neels, Maksym V. Kovalenko and Kostiantyn V. Kravchyk**

Supporting Information

Bilayer dense-porous LLZO membranes for high performance Li-garnet solid-state batteries

Huanyu Zhang, Faruk Okur, Claudia Cancellieri, Lars P.H. Jeurgens, Annapaola Parrilli, Dogan Tarik Karabay, Martin Nesvadba, Sunhyun Hwang, Antonia Neels, Maksym V. Kovalenko and Kostiantyn V. Kravchyk**

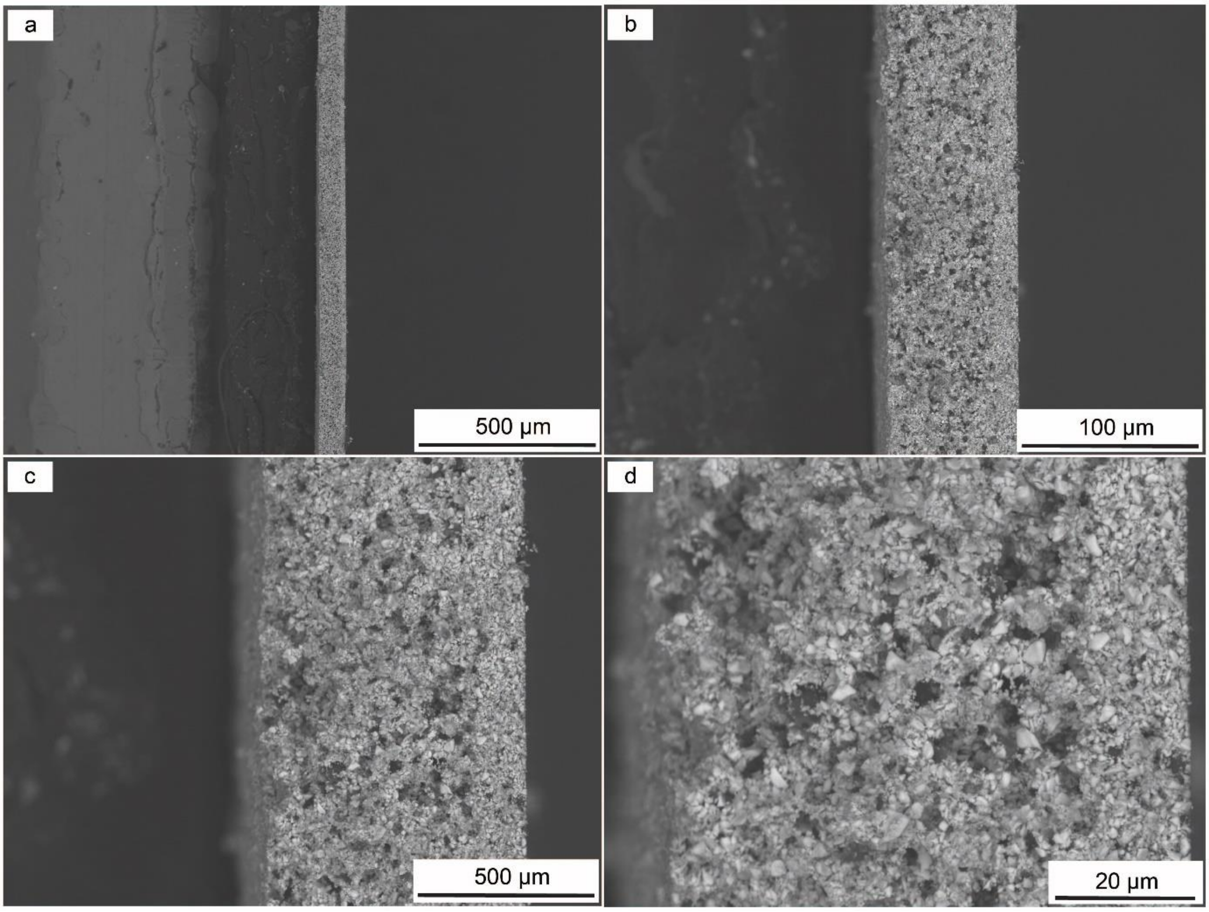

Figure S1. Cross-section SEM images of dense/porous bilayer membrane after de-binding.

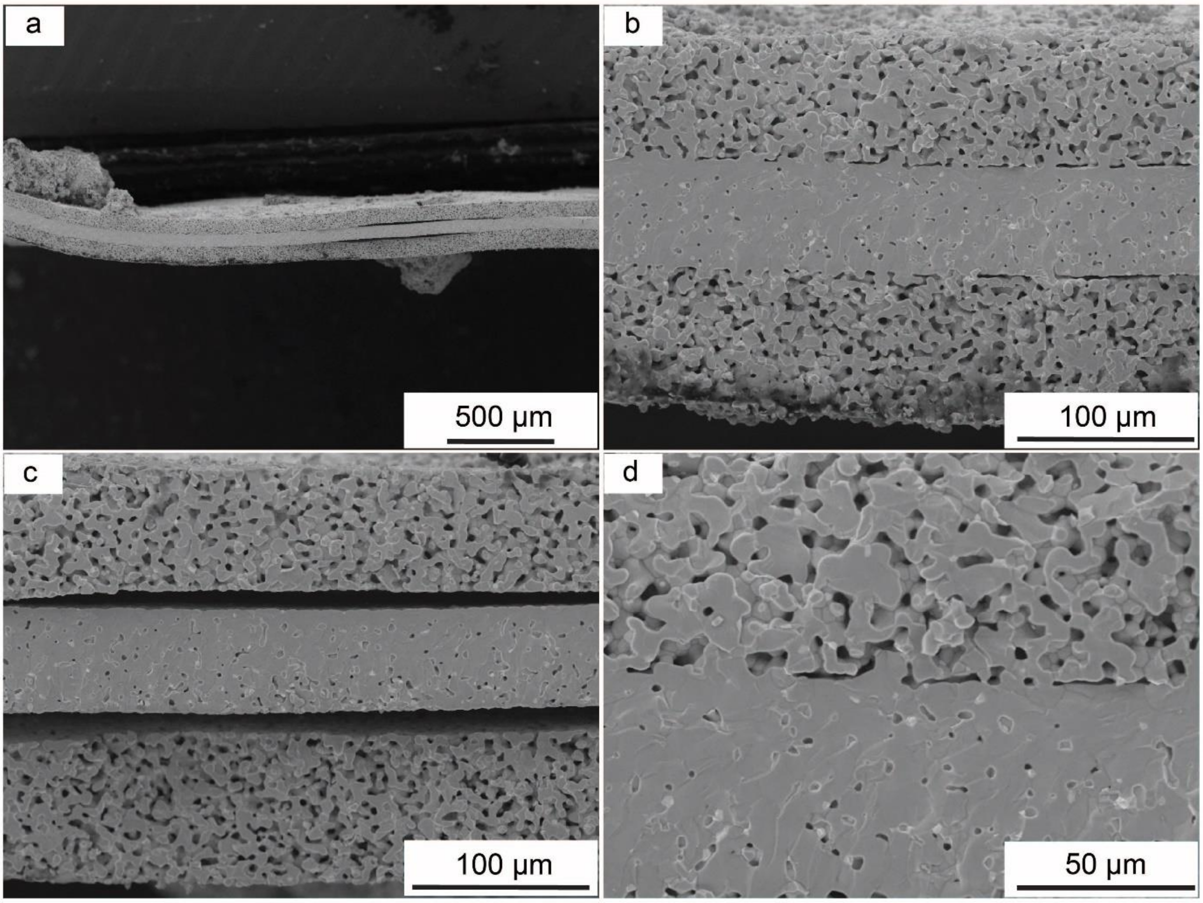

Figure S2. Cross-section SEM images of porous/dense/porous three-layer LLZO membranes after sintering. The membranes were prepared using previously reported methodology based on compressing of as prepared tape-casted porous and dense LLZO tapes.

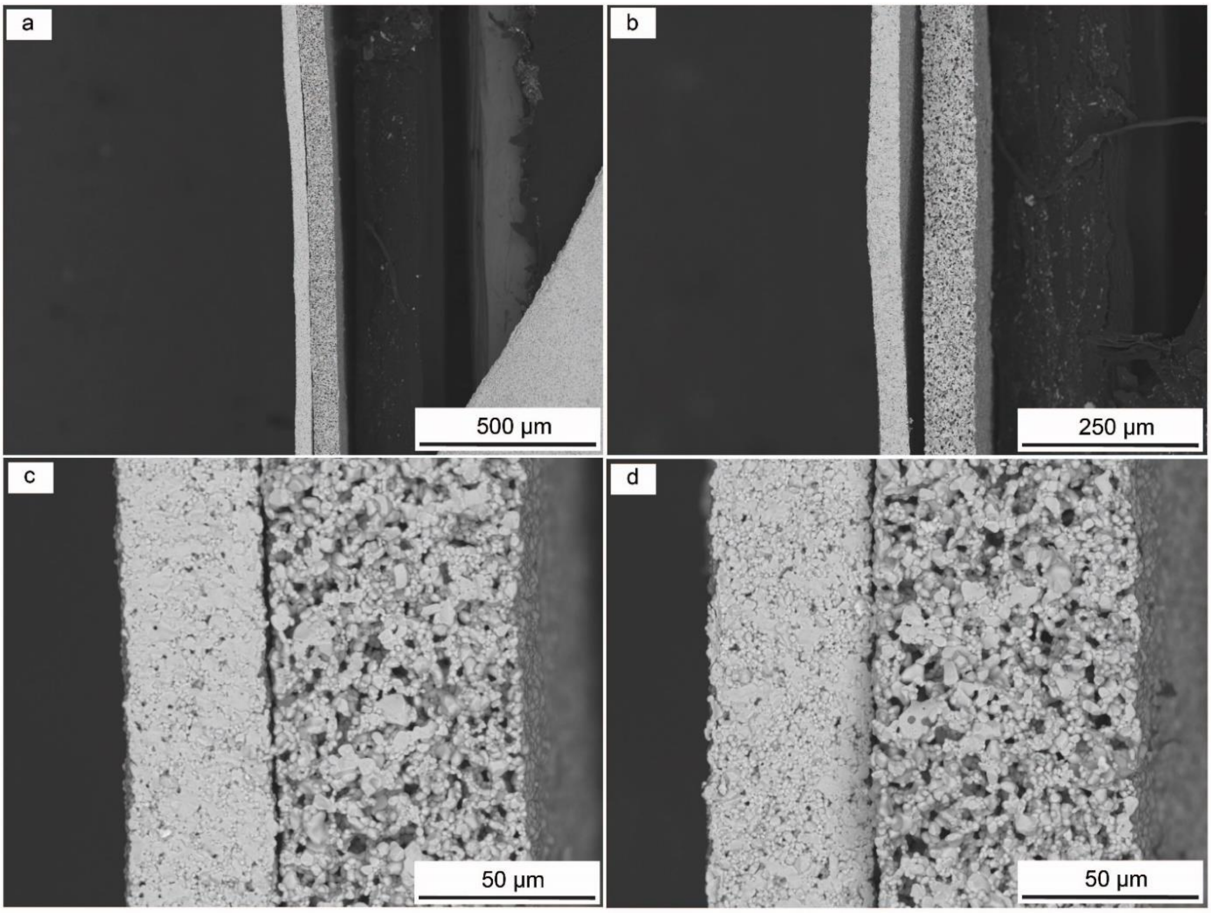

Figure S3. Cross-section SEM images of dense/porous two-layer LLZO membranes after sintering. The membranes were prepared by co-sintering of porous and dense LLZO tapes after de-binding.

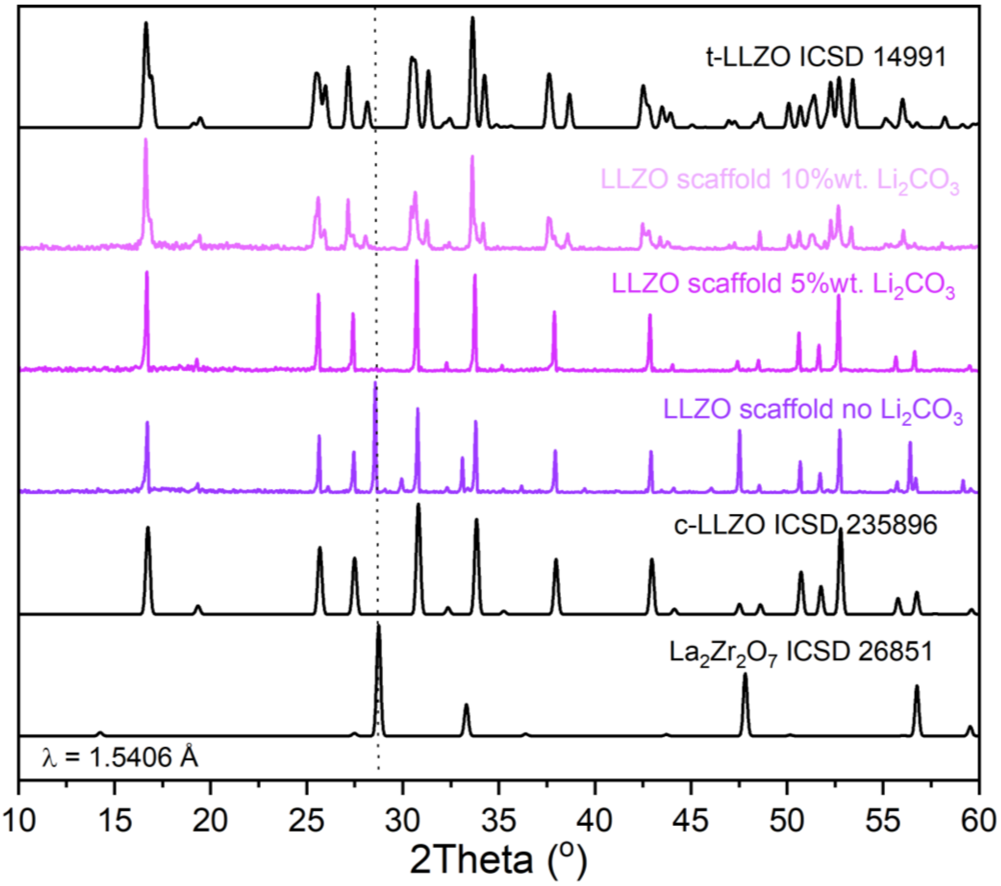

Figure S4. PXRD of sintered LLZO membranes, which were prepared using different amount of Li_2CO_3. XRD patterns of $La_2Zr_2O_7$ (ICSD 26851), cubic-LLZO (ICSD 235896) and tetragonal-LLZO (ICSD 14991) structures are given for comparison.

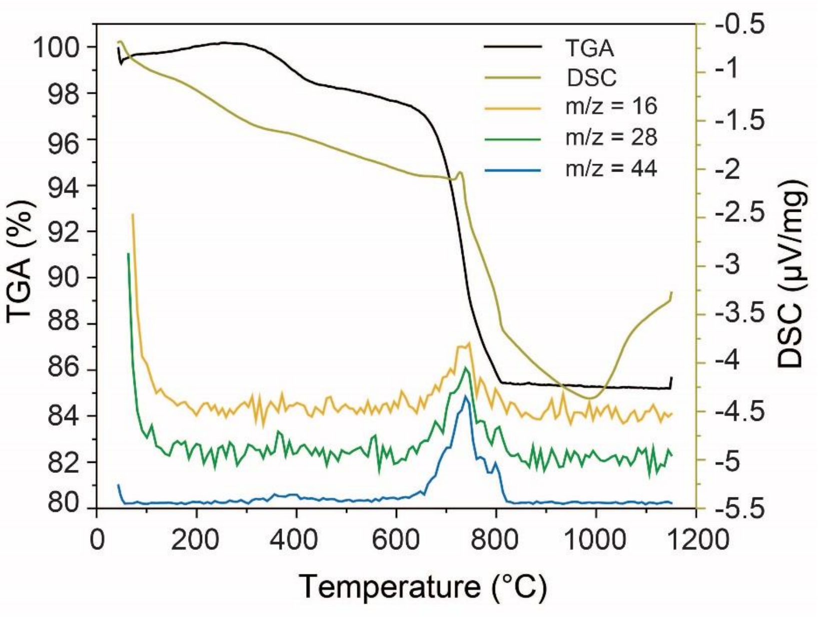

Figure S5. TGA-DSC-MA measurements of tape-casted LLZO membrane (before de-binding and sintering) performed at heating rate of 5 °C under Ar flow (40 mL min^{-1}).

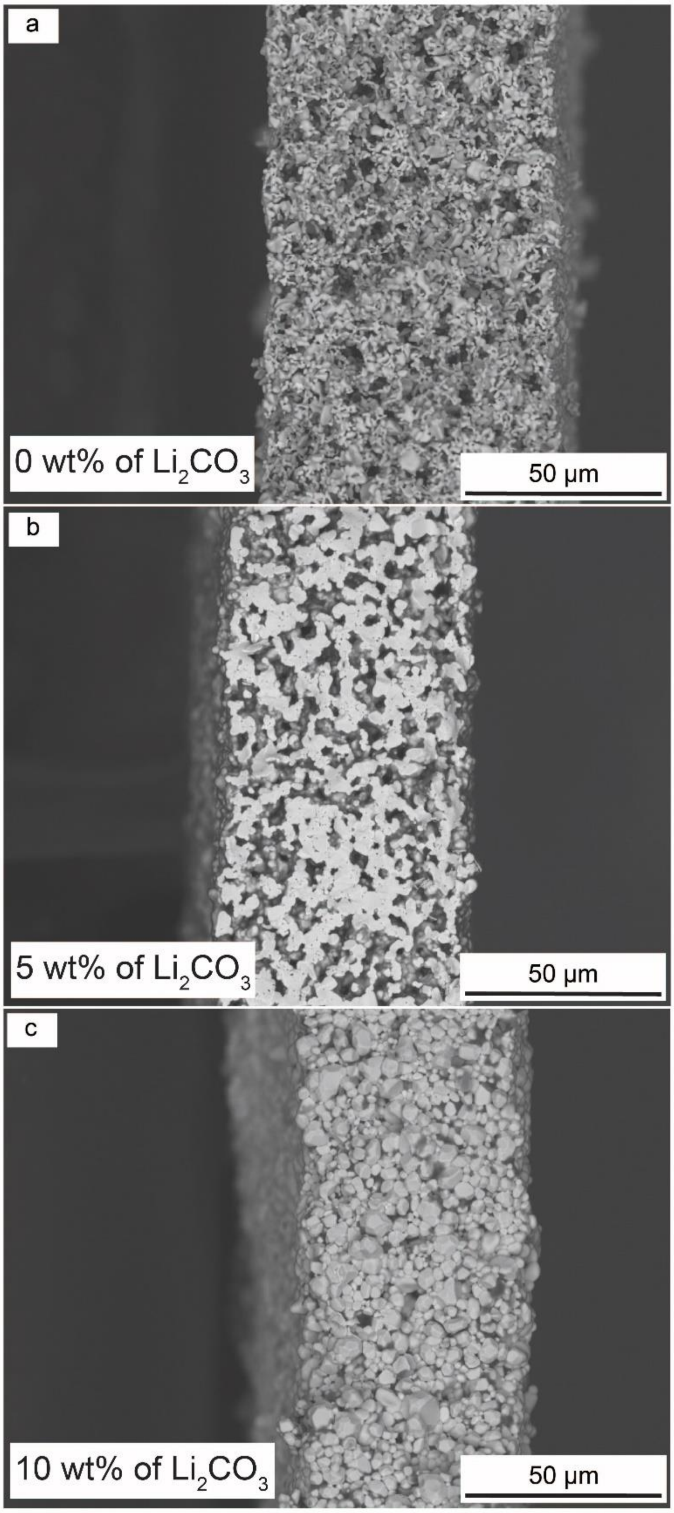

Figure S6. Cross-section SEM images of porous LLZO membranes after sintering, which were prepared using different amount of Li_2CO_3: 0 wt% Li_2CO_3 (a); 5 wt% Li_2CO_3 (b); 10 wt% Li_2CO_3 (c).

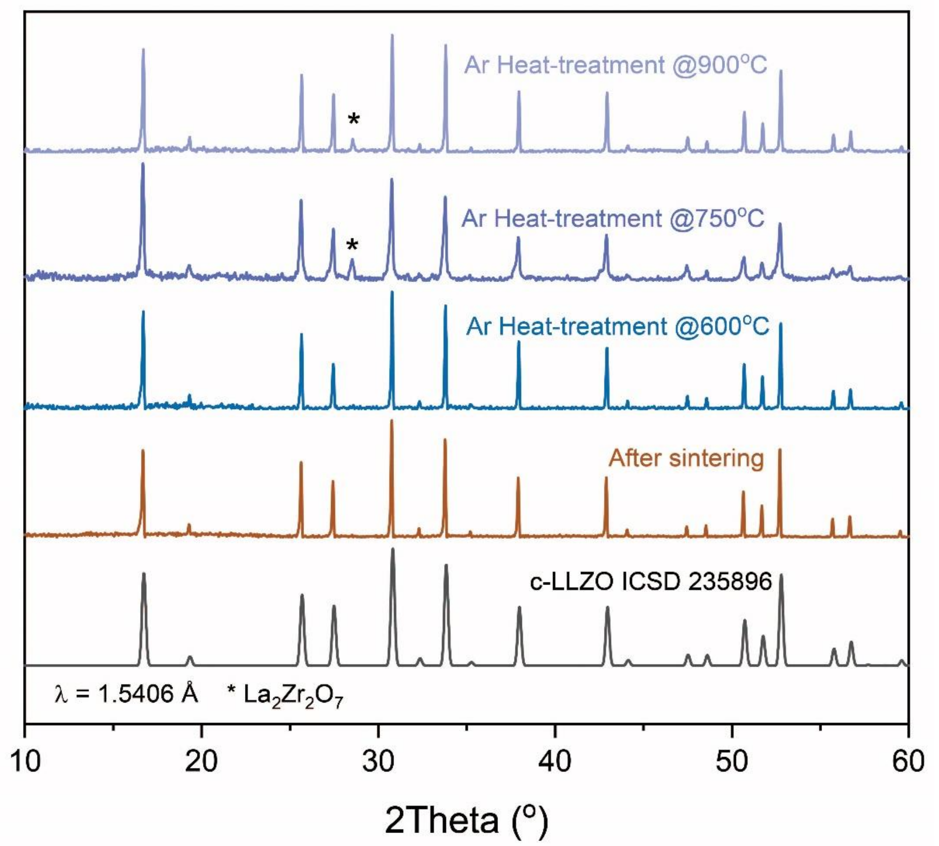

Figure S7. PXRD patterns of sintered LLZO membranes, which were additionally heat-treated under Ar atmosphere at 600 °C, 750 °C and 900 °C for 1h. PXRD pattern of non-heat-treated sintered LLZO membrane and XRD pattern of cubic-LLZO structure (ICSD 235896) are given for comparison.

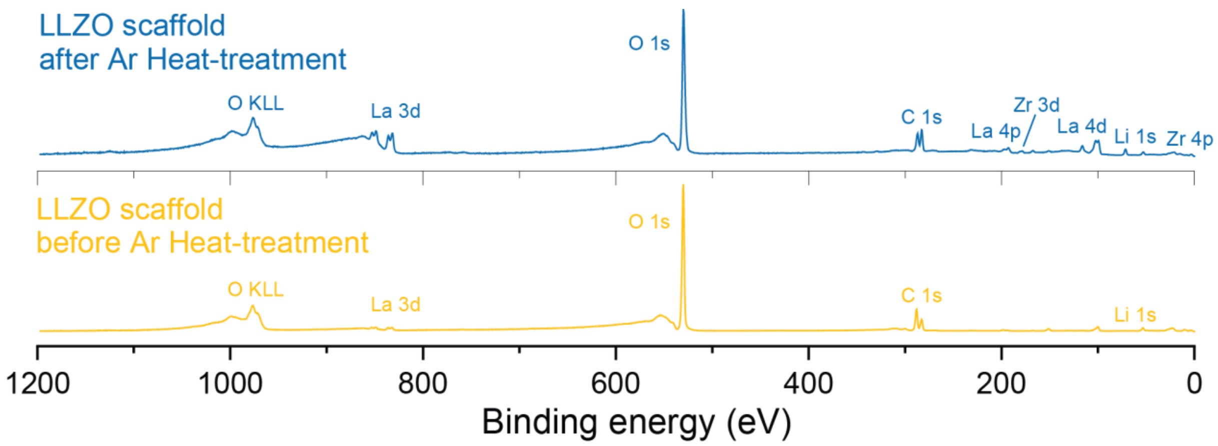

Figure S8. XPS surveys of sintered LLZO membrane before and after Ar heat-treatment (600 °C, 1h). The surveys were background corrected using a universal Tougaard cross-section for inelastic scattering.

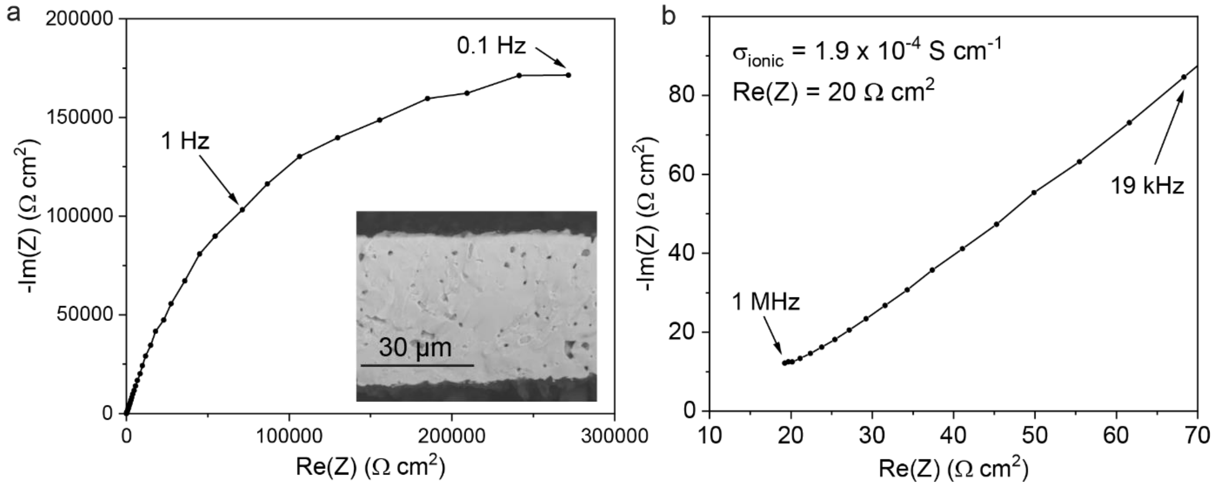

Figure S9. Impedance spectrum of dense LLZO membranes shown in different scales (inset: cross-section SEM image of dense LLZO membrane). The ionic conductivity of LLZO membrane (σ = 1.9×10^{-4} S cm^{-1}) was calculated from Re(Z) value of 20 Ω cm^2 (bulk and grain boundary resistance), LLZO thickness (38 µm) and the diameter of symmetrical thermally evaporated 50 nm Au electrodes (4 mm).

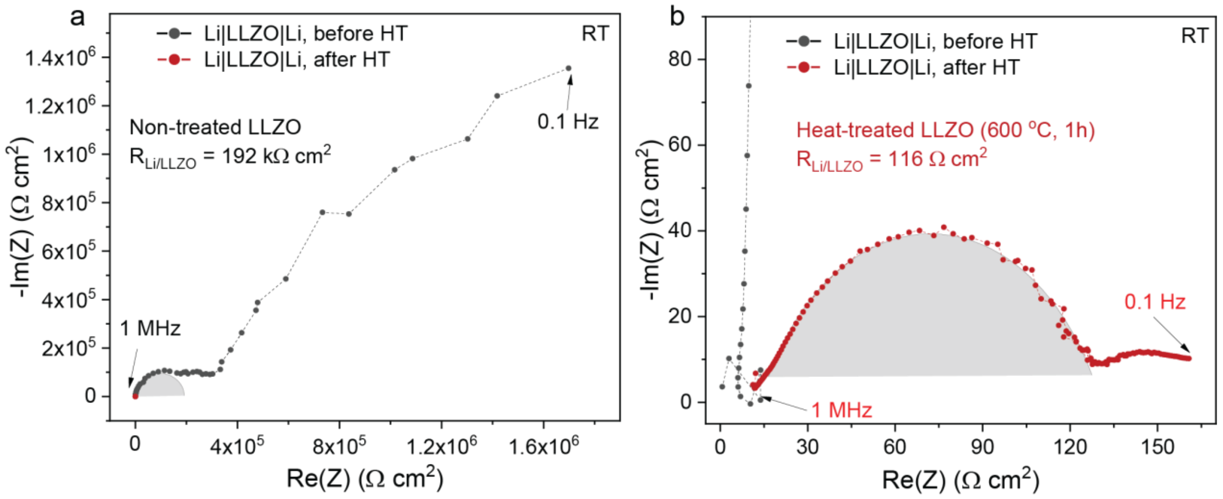

Figure S10. Impedance spectra of Li/LLZO/Li symmetrical cells assembled with non-heat-treated and heat-treated (600 °C;1h; Ar atmosphere) LLZO membranes.

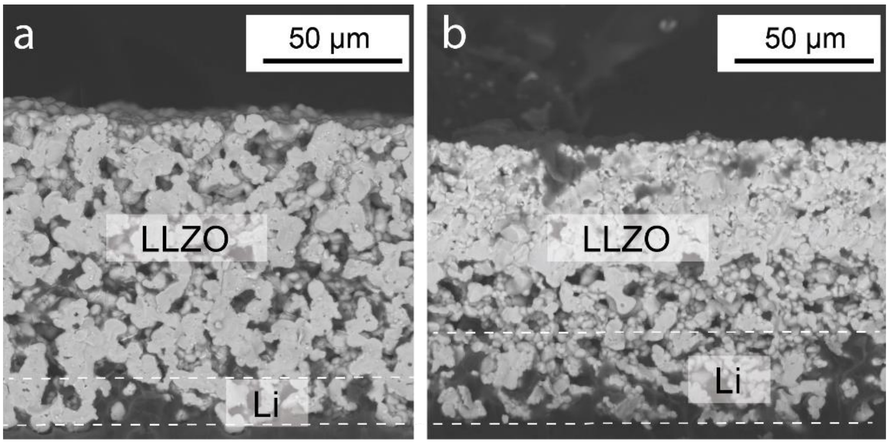

Figure S11. The comparison of cross-sectional SEM images of porous LLZO membranes after (a) isostatic pressing of metallic Li and (b) following Li plating in Li/LLZO/Li symmetric cell configuration at a current density of 0.1 mA cm^{-2}.

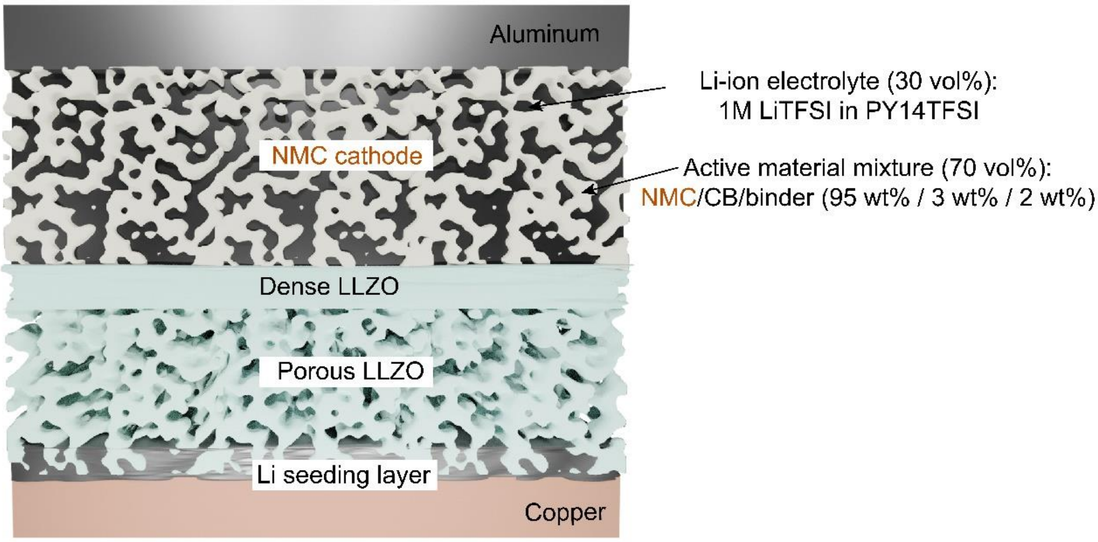

Figure S12. Schematics of the cell considered in this work for assessing the energy densities of Li-garnet SSBs.

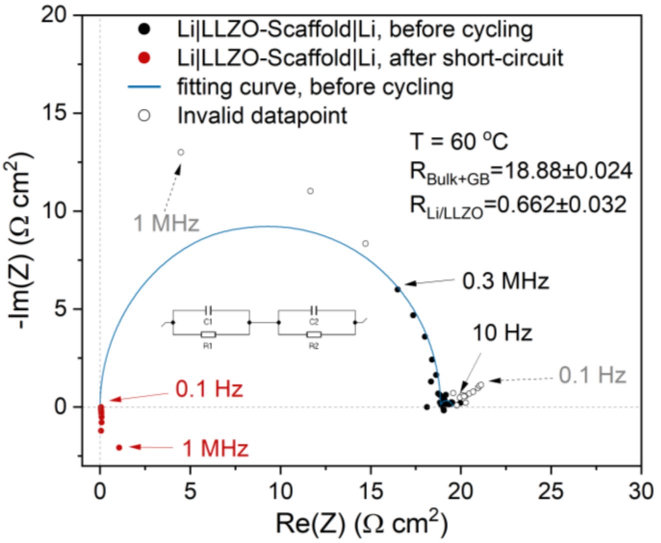

Figure S13. Impedance spectra of Li/porous LLZO membrane/Li symmetrical cell shown on **Figure 6a, b** before and after short-circuit. The thickness and the porosity of the membrane were *ca*. 60 µm and 55%, accordingly. Open cycles represent invalid data points due to the inaccuracy of EIS measurements with the VMP-3 instrument (Biologic) at high frequencies of > 100 kHz when the resistance of the measured sample is less than 10 kΩ.

Table S1. The porosity analysis of the porous layer in bilayer LLZO membrane performed using GeoDict software.

Size of sample	478 × 306 × 20 µm
Overall porosity	52.6559 %
Open porosity	52.6431 %
Closed porosity	0.0127686 %
Through porosity	52.6262 %
Dead-end porosity	0.016908 %
**Open porosity ratio**	**99.9757 %**
**Through porosity ratio**	**99.9436 %**

Table S2. Parameters used for the calculations of energy density.

**Parameters**	**Value**
Density of NMC811	4.77 g cm^{-3}
Loading of NMC811	3 mAh cm^{-2} (16.67 mg cm^{-2})
Density of Carbon	1.8 g cm^{-3}
Density of PVDF	1.68 g cm^{-3}
Thickness of cathode	57 µm
Average potential of NMC811 cathode	3.8 V *vs.* Li^+/Li
Theoretical capacity of NMC811 cathode	180 mAh g^{-1}_{NMC}
Thickness of Al foil	16 µm
Density of Al	2.7 g cm^{-3}
Thickness of Cu foil	12 µm
Density of Cu	8.96 g cm^{-3}
Density of LLZO solid-state electrolyte	5.1 g cm^{-3}
Density of LPS solid-state electrolyte	2 g cm^{-3}
Density of LiTFSI-Pyr14TFSI ionic liquid electrolyte	1.7 g cm^{-3}
Areal weight of packaging foil	10.5 mg cm^{-2}
